# Meetings of Societies

**Published:** 1949-10

**Authors:** 


### Meetings of Societies

### BRISTOL MEDICO-CHIRURGICAL SOCIETY.

Programme for 1949–50: each night 8.15 p.m.

October 12th. Annual General Meeting. Dr. Orr Ewing: President's Address.

November 9th. Clinical Meeting, Bristol General Hospital.

December 14th. Drs. Smart and Apley, "**Medicine in America**".

1950. January 11th. Professor R. C. Browne, "**The Work of an Industrial Health Department**".

February 8th. Rt. Hon. Lord Moran, *M.C.*, "**National Service Army**".

### ROYAL SOCIETY OF MEDICINE: SECTION OF PÆDIATRICS

THIS section of the Royal Society of Medicine visited Bristol on June 11th for its annual provincial meeting. There were 120 visitors. The clinical programme opened with a visit to the Premature Baby and Neonatal Departments at Southmead Hospital, where the technical aspects and problems of the departments were considered and expecially the improved methods of treatment of prematures. Emphasis was given to the care of the smaller prematures. The lay-out of a properly equipped "hot" room was demonstrated, and the associated series of "cooling" rooms were inspected and the new types of cots were displayed. Methods of controlling temperature, humidity and infection were discussed. Following this there was a short debate on obstetric-pædiatric relationships in a maternity department, it being pointed out that there was great scope for such teamwork. In the afternoon the Children's Hospital continued the programme with about forty clinical cases. The film of the Southmead "Good" quadruplets (from birth to ten months) was shown, more particularly to illustrate the progress of motor activity, emotional and character emergence, and individualised development.

### BATH CLINICAL SOCIETY, 1949–50.

*President:* W. Love. *Secretary:* A. Daunt Bateman. *Treasurer:* E. Scott-White. *Committee:* T. Cotter-Craig, J. M. Sanson, R. H. G. H. Denham.

Meetings first Thursday in each month at the Royal United Hospital at 8.15 p.m.

November 4th, 1949. Dr. G. D. Kersley, **"Impressions of Rheumatology in America".**

Annual General Meeting, May 5th, 1950.

### CORNWALL CLINICAL SOCIETY

IT has been agreed at a meeting of the staffs of the Royal Cornwall Infirmary and the Camborne-Redruth Hospital to amalgamate their respective Medical Societies under the title of the Cornwall Clinical Society. Meetings will be held monthly throughout the year alternately at the Royal Cornwall Infirmary and at the Camborne-Redruth Hospital; the meetings at the Royal Cornwall Infirmary will be on the first Thursday of the month, and at Redruth on the first Friday, in both cases at 8.15 p.m. Rules for the conduct of the new Society were drawn up and approved unanimously. The first President of the newly-constituted Society is Mr. M. R. Sheridan, F.R.C.S., and the joint Hon. Secretaries are Drs. L. W. Hale and F. D. M. Hocking. At the June meeting, the retiring President, Dr. F. D. M. Hocking, gave an account of a number of post-mortem examinations in which the apparently obvious cause of death was not, in fact, the true cause, emphasizing that a post-mortem examination must be complete in all cases.

### DEVON AND EXETER MEDICO-CHIRURGICAL SOCIETY.

March 3rd, 1949. Dr. W. R. Bett: **"Drink, Drugs and Doctors".** Halsted was socially a timid person, but professionally formidable. He introduced rubber gloves as a means of protecting his theatre-sister's hands from Lister's mercuric lotion, then in general use. As a result of experiments with cocaine he became an addict and was only rescued with difficulty. Roderigo Lopez, first house surgeon at Barts, was accused of poisoning Queen Elizabeth, and duly hanged. The lecturer referred to the poisoners Palmer and Crippen, and the effects of opium addiction in Crabbe, Francis Thompson Coleridge, de Quincey, Baudelaire and others.

PLYMOUTH MEDICAL SOCIETY PROGRAMME FOR 1950.

January 13th, at 8.30 p.m. Professor C. Bruce Perry: "**The Diagnosis and Treatment of Acute Rheumatism**".

February 1st, Mount Gold, at 5.30 p.m. Orthopœdic demonstration.

February 17th, at 8.30 p.m. Professor D. R. MacCalman: "**The Psychological Aspects of Medical Practice**".

March 3rd, at 8.30 p.m. Dr. G. S. Todd: "**Streptomycin and Pulmonary Tuberculosis**".

March 24th, at 8.30 p.m., Greenbank. Clinical Evening.

BRITISH MEDICAL ASSOCIATION

BATH, BRISTOL AND SOMERSET BRANCH

November 2nd. 8.30 p.m. at Bristol University (Royal Fort). Annual General Meeting. Presidential Address: "**The Forgotten Inventor**".

